# Occurrence and risk assessment of okadaic acid, dinophysistoxin-1, dinophysistoxin-2, and dinophysistoxin-3 in seafood from South Korea

**DOI:** 10.1007/s11356-023-31568-4

**Published:** 2023-12-26

**Authors:** Jong Bin Park, Solyi Cho, Sang Yoo Lee, Su Mi Park, Hyang Sook Chun

**Affiliations:** Food Toxicology Laboratory, School of Food Science and Technology, Chung-Ang University, Anseong, 17546 South Korea

**Keywords:** Diarrheic shellfish poisoning, Korean seafood, OA-group toxin, Occurrence, Risk assessment, Bivalves

## Abstract

The okadaic acid (OA)-group toxins, including OA, dinophysistoxin-1 (DTX1), dinophysistoxin-2 (DTX2), and dinophysistoxin-3 (DTX3), cause diarrheic shellfish poisoning in humans. To manage OA-group toxins more strictly, Korean regulations were recently revised to consider OA, DTX1, DTX2, and DTX3 combined. Thus, our study characterized the occurrence of OA, DTX1, DTX2, and DTX3 in seafood distributed across South Korea, and a risk assessment of seafood consumption was conducted. Two hundred and seventeen samples from 16 bivalve and 7 non-bivalve species collected from three representative coastal areas in 2021 were analyzed via liquid chromatography-tandem mass spectrometry. OA, DTX1, and DTX3 were detected in 2.3%, 4.1%, and 9.2% of the examined samples, with positive mean levels of 11.3, 16.4, and 40.9 µg/kg, respectively. DTX2 was not detected in any of the samples. At least one OA-group toxin was detected in the bivalve samples, including blood clams, pan shells, hard clams, mussels, and scallops, whereas none were detected in non-bivalves. The estimated acute exposure to OA-group toxins through the intake of seafood in the Korean population and consumer groups was low, ranging from 24.7 to 74.5% of the recommended acute reference dose (ARfD) of 0.33 μg OA equivalents/kg body weight. However, for the scallop consumers aged 7–12 years, acute exposure to OA-group toxins exceeded the ARfD, indicating a possible health risk. These results suggest that including DTX3 in the new regulatory limits is appropriate to protect Korean seafood consumers from exposure to OA-group toxins.

**Supplementary Information:**

The online version contains supplementary material available at 10.1007/s11356-023-31568-4.

## Introduction

The okadaic acid (OA)-group toxins encompasses OA and its analogs dinophysistoxin-1 (DTX1), dinophysistoxin-2 (DTX2), and dinophysistoxin-3 (DTX3). OA, DTX1, and DTX2 are synthesized by toxin-producing microalgae, namely *Dinophysis acuminata* (OA, DTX1), *D*. *ovum* (OA), *Prorocentrum lima* (OA, DTX1), *D*. *acuta* (OA, DTX2), and *D*. *caudata* (OA, DTX2), and tend to accumulate in the hepatopancreas of various species of filter-feeding mollusks, including mussels, clams, oysters, and scallops (Dominguez et al. 2010; EFSA 2008; EFSA 2021). Therefore, the consumption of contaminated shellfish can cause diarrheic shellfish poisoning (DSP), which is characterized by diarrhea, nausea, vomiting, and abdominal pain (Larsen et al. 2007; Lee et al. 2016).

The OA-group toxins are polyether compounds that have lipophilic properties. They are stable between − 20 and 80 °C but are substantially degraded above 100 °C (FAO 2004; McCarron et al. 2008; Yasumoto and Murata 1993). The main representative OA-group toxin is OA, a linear polyketide with a molecular weight of approximately 800 Da that contains several ether rings and a terminal carboxylic acid moiety (Valdiglesias et al. 2013). DTX3 is thought to be a metabolic product of OA, DTX1, and DTX2, which is produced within the shellfish itself (Yanagi et al. 1989). Specifically, DTX3 is a heterogeneous group of fatty acid ester derivatives of the OA, DTX1, and DTX2 toxins, in which the 7-hydroxy position is esterified with an acyl chain 14–22 carbons in length (EFSA 2008). However, DTX3 is deacylated to the parent toxin (i.e., OA, DTX1, or DTX2) under alkaline conditions, at elevated temperatures, or in the presence of enzymes such as lipase and cholesterol esterase (EFSA 2008).

Sea temperature may be one of the most important environmental factors influencing the distribution and toxin content of *Dinophysis* species, as plankton growth rates generally increase with increasing temperature within a certain range (Borges et al. 2022; Griffith and Gobler 2020; Hallegraeff 2010). This has led to the assumption that the occurrence of DSP and the synthesis of lipophilic toxins by *Dinophysis* species also depend on the environmental temperature (Kamiyama 2010). The sea temperature off the coast of Korea is steadily rising each year. The average seawater temperature from 1968 to 2020 was 16.8 °C in the East Sea, 18.7 °C in the South Sea, and 15.0 °C in the West Sea. However, the annual temperatures of the East Sea, South Sea, and West Sea in 1968 and 2020 have increased by 1.9, 1.3, and 0.9 °C, respectively (Korea Meteorological Administration 2021). Previous studies identified OA-group toxins in the South Sea, which is the water body with the highest temperature off the Korean coast (Park et al. 2019). Therefore, based on the increasing sea temperatures and the report by Kamiyama (2010), OA and its DTX analogs may also be found in the East Sea.

OA-group toxins are known to inhibit protein (serine and threonine) phosphatases (PPs), which is their mechanism of action. The inhibition of type 1 PPs (PP1) and type 2A PPs (PP2A) is of particular significance. After ingestion, OA-group toxins inhibit PPs in enterocytes, destabilizing sodium concentration, which leads to diarrhea-like symptoms due to osmotic pressure differences (Munday 2013; Tripuraneni et al. 1997). DTX3 cannot bind to PPs and is therefore less toxic than the other OA-group toxins. However, when humans consume seafood, certain hydrolases can convert DTX3 into its corresponding precursors (OA, DTX1, or DTX2) and increase its toxicity. Therefore, additional efforts are needed to manage DTX3 and the free forms of OA-group toxins. However, no chronic toxicity/carcinogenicity studies have been conducted for OA and its analogs (EFSA 2008).

A tolerable daily intake of OA-group toxin has yet to be established because data on the chronic effects of OA toxins are insufficient. However, an acute reference dose (ARfD) has been established based on the acute effects of human exposure cases. The Joint FAO/IOC/WHO ad hoc Expert Consultation suggested a provisional ARfD of 0.33 µg OA equivalents (eq.)/kg body weight (bw) (FAO/IOC/WHO 2004). The European Food Safety Authority (EFSA) established toxic equivalency factor (TEF) values of 1 for OA, 1 for DTX1, and 0.6 for DTX2 (EFSA 2008). The FAO/WHO (2016) reviewed those TEFs and proposed changing the TEF of DTX2 to 0.5 but leaving that of OA and DTX1 unchanged (FAO/WHO 2016). For DTX3, the TEF values are equal to those of the corresponding unesterified toxins, including OA, DTX1, and DTX2 (EFSA 2008; FAO/WHO 2016).

To protect consumers from OA-group toxins, the governments of the European Union, the USA, Canada, and Japan have established maximum limits of 160 µg OA equivalent (eq.)/kg in the edible parts of live bivalves; this includes a combination of free OA, DTX1, and DTX2, and their acyl-esters (DTX3) (EFSA 2021; FDA 2019a; MHLW 2015). Regarding the Codex Alimentarius and the Australian government, OA, DTX1, and DTX2 are managed based on a maximum allowable limit of ≤ 200 µg OA eq./kg (CODEX 2015; Food Standards Australia New Zealand 2021). In New Zealand, OA, DTX1, DTX2, and pectenotoxins 1 and 2 are managed based on a maximum allowable limit of ≤ 160 µg OA eq./kg (New Zealand Ministry for Primary Industries 2020). In Korea prior to 2023, only OA and DTX1 were managed based on a maximum limit of ≤ 160 µg OA eq./kg (MFDS 2016).

Data on the occurrence of OA toxins including OA, DTX1, DTX2, and DTX3 in the seafood consumed in Korea are currently insufficient, and risk assessment studies based on these data are needed. Additionally, it is important to review the current strategies for the regulation of OA-group toxins by determining the risk level of OA exposure associated with the consumption of marine products by the Korean population. Therefore, in the present study, we aimed to investigate the occurrence of OA, DTX1, DTX2, and DTX3 in seafood (16 bivalves and 7 non-bivalves) commonly consumed in South Korea, after which we conducted a risk assessment of seafood consumption based on the acquired data.

## Materials and methods

### Samples

Two hundred and seventeen seafood samples were collected, encompassing 16 bivalve and seven non-bivalve species from across South Korea. These seafood samples were selected to be representative of the Korean population’s seafood intake and the previous detection history of OA-group toxins (Supplementary Table S1). Among non-bivalves, flatfish was selected to investigate its potential as toxin vector and threat to public health, considering its feeding behavior of consuming bivalves (Mafra et al. 2014; Sipia et al. 2000). The bivalve samples included 13 blood clams (*Anadara broughtonii*), 13 blood cockles (*Tegillarca granosa*), 21 clams (*Ruditapes philippinarum*), 18 fan shells (*Atrina pectinata*), 26 hard clams (*Mercenaria mercenaria*), 15 Japanese cockles (*Fulvia mutica*), 11 *Mactra* sp. (9 M. *quadrangularis* and 2 M*. chinensis*), 21 mussels (5 *Mytilus coruscus* and 16 M. *galloprovincialis*), 9 oysters (*Crassostrea gigas*), 23 scallops (14 *Mizuhopecten yessoensis* and 9 *Argopecten irradians*), 1 soft-shell clam (*Mya arenaria*), 1 sunset clam (*Megangulus venulosus*), and 10 surf clams (*Spisula sachalinensis*). The non-bivalve samples included 9 abalones (*Haliotis discus hannai*), 6 conches (1 *Turbo cornutus* and 5 *Rapana venosa*), 4 crabs (*Portunus trituberculatus*), 6 flatfishes (*Paralichthys olivaceus*), 4 sea cucumbers (*Apostichopus japonicus*), and 6 sea urchins (*Heliocidaris crassispina*). The collected samples were used after identifying each species based on morphological characteristics and/or species-specific genetic marker analysis by Professor Kwang-Sik Choi at Jeju National University.

Sampling was conducted from April to October 2021, with most samples being acquired in the summer (June–August). Fifty-two samples (24%) were collected in April, 41 (19%) in May, 38 (18%) in June, 39 (18%) in July, 30 (14%) in August, 11 (5%) in September, and 6 (3%) in October. The samples were collected from as many sites as possible to assess the variations in toxin levels according to the marine species of each harvested area. The samples obtained from each of the sampling sites were as follows: 15 samples in the East Sea (7%), 73 in the West Sea (34%), 97 in the South Sea (45%), 15 in China (7%), nine in Japan (4%), and eight in Russia (4%).

More than 200 g of each sample was collected to ensure representative sampling and more than 1 kg of live shellfish was purchased online. The shells of the live shellfish, which were covered with sand, were cleaned under running water and opened with a knife being careful not to damage the tissues or internal organs; the flesh was then separated. In the case of flatfish, only the edible part (i.e., the flesh) was used after removal of inedible parts such as the head, intestines, and bones. The separated samples were cleaned with distilled water to remove foreign materials and drained for 5 min in a sieve. The samples were then homogenized in a blender for 1 min at room temperature. The homogenized samples were stored at − 18 °C and thawed before use.

### Chemicals and reagents

HPLC-grade water, methanol (MeOH), and acetonitrile (MeCN) were purchased from Burdick & Jackson (Morris Plains, NJ, USA). Liquid chromatography-mass spectrometry (LC-MS)-grade formic acid and ammonium formate (> 99.0%) were purchased from Thermo (Waltham, MA, USA) and Sigma (St. Louis, MO, USA), respectively. Standard stock solutions of OA-group toxins in MeOH (OA, 8.37 mg/L; DTX1; 8.52 mg/L; and DTX2, 3.78 mg/L) were purchased from the National Research Council Canada (NRC) (Halifax, NS, Canada). One of the compounds from the DTX3 complex, 7-O-palmitoyl OA, is commercially available as DTX3a and was purchased from CIFGA (Lugo, Spain). Working standard stock solutions of 1 mg/L were prepared by dilution in MeOH and were stored at − 18 °C. The working standards were left at room temperature before use. Working standards at a concentration of 80, 60, 40, 20, 10, and 2 µg/L were obtained by appropriate dilution in MeOH. The samples were then purified using Waters Sep-Pak C18 solid-phase extraction (SPE) cartridges (Milford, MA, USA) on a 12-port vacuum manifold (Supelco, Bellefonte, PA, USA).

### Toxin extraction and cleanup

Toxin extraction was performed as described by the European Union Reference Laboratory for Marine Biotoxins with slight modifications (EURLMB 2015). Briefly, a sub-sample (2.000 ± 0.005 g) of tissue homogenate was weighed into a 50-mL conical tube. After adding 9 mL of MeOH, the samples were homogenized for 3 min using an Ultra-Turrax homogenizer (IKA, Staufen, Germany). The mixture was centrifuged (2,000 × *g*, 10 min), and the supernatant was transferred to a 25-mL graduated cylinder. The entire extraction procedure was performed twice. The final volume was adjusted to 20 mL with MeOH. Two milliliters of supernatant were transferred to 5-mL glass vials to analyze free OA, DTX1, and DTX2 and their acylated ester DTX3. To determine the total content of OA group toxins including DTX3, an alkaline hydrolysis is necessary from methanolic extract prior to LC-MS/MS analysis with the aim of converting any acylated esters of OA and/or DTXs to the parent OA and/or DTX1 or DTX2 toxins (EURLMB 2015). The samples were subjected to alkaline hydrolysis by adding 250 µL of 2.5 N NaOH per vial. The vial lids were tightly closed, and the samples were hydrolyzed at 76 °C for 40 min. The samples were then allowed to cool to room temperature and neutralized by adding 250 µL of 2.5 N HCl. For free OA, DTX1, and DTX2 analyses, 2 mL of extract was defatted without prior hydrolysis. For defatting, 2.5 mL of hexane was added to both hydrolyzed and unhydrolyzed solutions and vortexed for 30 s. After removing the hexane layer, 2.5 mL of water was added to dilute the solution. SPE cleanup was conducted using a Sep-Pak C18 cartridge (Lee et al. 2022). The cartridge was conditioned with 5 mL of MeOH and 5 mL of water. After conditioning, the diluted extract was loaded. The empty vial was washed with 3 mL of 40% MeOH, and the wash liquid was also loaded on the cartridge to maximize recovery. The cartridge was washed with 3 mL water and 3 mL of 40% MeOH. Next, the material was eluted with 6 mL of MeOH. The eluted solutions were dried under a stream of nitrogen gas at 40 °C and redissolved in 2 mL MeOH. The final redissolved extract was filtered through a 0.22 µm PVDF membrane filter prior to liquid chromatography-tandem mass spectrometry (LC–MS/MS) analysis.

### LC–MS/MS analysis

LC-MS/MS analysis was conducted with a setup consisting of an Agilent 1290 Infinity II LC systems coupled to Agilent 6470 triple quadrupole LC/MS instruments (Agilent Technologies, Santa Clara, CA, USA). An Xbridge C18 column (4.6 × 100 mm, 3.5 µm) from Waters (Milford, MA, USA) was used for separation. The column oven was maintained at 40 °C, and the autosampler compartment was maintained at 15 °C. The injection volume was 5 µL and the flow rate was 0.4 mL/min. Both mobile phase A (water) and mobile phase B (acetonitrile-water 95:5, v/v) contained 2 mmol/L ammonium formate and 50 mmol/L formic acid. The elution gradient was started with 90% solvent A for 1 min. Solvent A was then decreased to 10% over 9 min and held for 3 min. Then, solvent A was increased to 90% over 2 min and held for 4 min to re-equilibrate the column. The entire analysis procedure was completed in 19 min. The analytes were detected using a triple quadrupole mass spectrometer (G6470B). Electrospray ionization in negative ion mode was used to detect the three toxins. Ultrapure nitrogen (purity 99.999%) was used as a collision gas. The optimized MS parameters were as follows: gas temperature, 305 °C; gas flow, 9 L/min; nebulizer, 30 psi; sheath gas temperature, 350 °C; sheath gas flow, 12 L/ min; and capillary voltage, − 4500 V. The precursor ion and product ion ([M-H]-) were confirmed for OA (803.5 m*/z* and 255 m*/z*, respectively), DTX1 (817.5 m*/z* and 563 m*/z*, respectively), and DTX2 (803.5 m*/z* and 225 m*/z*, respectively). The ion pair with the highest relative intensity was selected for quantification, whereas the ion pair with the second highest relative intensity was used as a qualifier ion for identification.

### Quantification of OA-group toxins

Individual OA-group toxins were quantified using the external matrix-matched standard calibration method (Lee et al. 2022). Individual toxin levels were calculated based on a calibration curve, and only results above the limit of detection (LOD) were subjected to further calculations. After hydrolysis, the content of each toxin was first calculated as the content of OA-group toxins in seafood samples. The level of DTX3, as esterified OA group toxins, was then calculated using the following formula: [amount of OA, DTX1, and DTX2 (with hydrolysis) − amount of OA, DTX1, and DTX2 (without hydrolysis)]. Afterward, the TEFs of OA, DTX1, and DTX2 were multiplied and summed to obtain the OA level (μg OA eq./kg). Our analyses were based on the TEFs proposed by FAO/WHO (2016).

### Method performance

Method performance was assessed based on the Food and Drug Administration (FDA) guidelines for the analysis of chemicals in food (FDA 2019b). The analysis method was validated using three matrices—mussel, clam, and flatfish—and the following factors were considered: linearity, LOD, limit of quantification (LOQ), accuracy, and precision. Linearity was confirmed through the calibration curve's coefficient of determination (*R*^2^) calculated from at least five points at a LOD range of 40 µg/kg. The obtained *R*^2^ value of > 0.99 was considered excellent. LOD and LOQ were calculated with the following formula using the slope (S) of the calibration curve and standard deviation (σ) of the replicate analysis (LOD = 3.3 × σ/S, LOQ = 10 × σ/S). Assay accuracy and precision were evaluated as the recovery value and relative standard deviation (RSD), respectively, by intra-day (*n* = 3) and inter-day (*n* = 3) replicate assays. The recovery test was performed at 100 µg/kg with three matrices.

### Statistical analysis

Student’s* t*-test or one-way analysis of variance followed by Duncan’s multiple range test were performed to test significant differences in OA-group toxin levels between samples, regions, or seasons (IBM SPSS Statistics 20, IBM Corporation, Armonk, NY, USA). For all analyses, *p* < 0.05 was considered statistically significant.

### Risk assessment of acute OA-group toxin exposure

Acute dietary exposure to bivalves and non-bivalves was assessed through a deterministic approach using the occurrence data derived from this study coupled with seafood consumption and body weight trends. Exposure assessment was performed and compared using two types of occurrence data: the sum of OA, DTX1, DTX2, and DTX3, and the sum of OA and DTX1, the latter of which is the previous Korean regulation for OA-group toxins in food. For occurrence level, 0 was used to replace the ND (non-detected) values in the lower bound (LB), whereas LOD was used to replace the ND result in the upper bound (UB) (GEMS/Food-Euro 1995). Consumption and body weight data were based on the 2016–2018 Korea National Health and Nutrition Examination Survey (KCDC 2020; KHIDI 2020). Acute dietary exposure assessments were based on the maximum toxin level in each sample from the monitoring data, as indicated in the “Guidelines for the Study of Dietary Intakes of Chemical Contaminants” published by the World Health Organization (WHO 1985). Based on these criteria, the 95th percentile of food consumption was used (WHO 1985). Additionally, for a more detailed assessment, our analyses were conducted according to the age of the population and consumer groups. Regarding population consumption, if the 95th percentile of food consumption was not attained because the consumption rate was < 5%, the consumption was calculated by multiplying the average value by 2.5 (WHO 1985). The body weight was defined as the average value of each group. The following equation was used for estimating toxin exposure:

1 $$\mathrm{Acute}\;\mathrm{dietary}\;\mathrm{exposure}\;\mathrm{value}\;\left(\mu\mathrm g\;\mathrm{OA}\;\mathrm{eq}/\mathrm{kg}\;\mathrm{bw}/\mathrm{day}\right)=\;\frac{95\mathrm{th}\;\mathrm{percentile}\;\mathrm{of}\;\mathrm{food}\;\mathrm{consumption}\;(\mathrm g/\mathrm{day})\times\mathrm{Highest}\;\mathrm{residue}\;\left(\mu\mathrm g\;\mathrm{OA}\;\mathrm{eq}/\mathrm{kg}\right)}{\mathrm{bodyweight}\;\left(\mathrm{kg}\right)}\times10^{-3}$$

Risk assessment was conducted by comparing the dietary exposure value with the ARfD values of 0.33 µg OA eq./kg bw/day from the FAO/IOC/WHO and 0.30 µg OA eq./kg bw/day from the EFSA. %ARfD > 100% is commonly recognized as a high-risk level. The following equation was used for estimating the %ARfD:

2 $$\%\mathrm A\mathrm R\mathrm f\mathrm D=\frac{\mathrm A\mathrm c\mathrm u\mathrm t\mathrm e\;\mathrm d\mathrm i\mathrm e\mathrm t\mathrm a\mathrm r\mathrm y\;\mathrm e\mathrm x\mathrm p\mathrm o\mathrm s\mathrm u\mathrm r\mathrm e\;\mathrm v\mathrm a\mathrm l\mathrm u\mathrm e\left(\mu\mathrm g\;\mathrm O\mathrm A\;\mathrm e\mathrm q/\mathrm k\mathrm g\;\mathrm b\mathrm w/\text{day}\right)}{\mathrm{ARfD}\left(\mu\mathrm g\;\mathrm O\mathrm A\;\mathrm e\mathrm q/\mathrm k\mathrm g\;\mathrm b\mathrm w/\text{day}\right)}\times100$$

## Results

### Performance criteria for the determination of OA-group toxins

To investigate the occurrence of OA-group toxins in seafood distributed in Korea, we first evaluated whether the utilized analytical method satisfies the performance criteria. Supplementary Table S2 summarizes the results of the intra- and inter-day precision of the experiments conducted using clam, flatfish, and mussel samples spiked with 100 µg/kg of different OA-group toxins. All *R*^2^ values exceeded 0.997. The LOD and LOQ ranges in the three matrices were 0.5–2.1 µg/kg and 1.6–6.2 µg/kg for OA, 0.5–2.6 µg/kg and 1.4–7.8 µg/kg for DTX1, and 0.4–2.6 µg/kg and 1.2–7.8 µg/kg for DTX2, respectively. The intra- and inter-day accuracies expressed as recovery rates evaluated in the three matrices were 85.7–105.7% for OA, 82.9–99.2% for DTX1, and 89.8–109.3% for DTX2. The intra- and inter-day precisions expressed as RSD (%) evaluated in the three matrices were 3.9–8.6% for OA, 3.8–14.6% for DTX1, and 2.0–17.0% for DTX2.

### Occurrence of OA-group toxins in Korean seafood

Among the 217 samples collected, OA was detected in five of them (detection rate, 2.3%; positive mean level, 11.3 µg/kg), DTX1 was detected in nine (detection rate, 4.1%; positive mean level, 16.4 µg/kg), DTX3 was detected in 20 (detection rate, 9.2%; positive mean level, 40.9 µg/kg), and DTX2 was not detected (Table 1). According to the TEF of the OA-group toxins, the positive mean level of total OA-group toxins was 50.23 µg OA eq./kg. The samples in which OA-group toxins were most detected were Korean mussels (*M. coruscus*, five cases) and clams (*R. philippinarum*, five cases). However, OA-group toxins were also detected in bay scallops (*A*. *irradians*, one case), fan shells (*A*. *pectinata*, two cases), blood clams (*A*. *broughtonii*, two cases), hard clams (*M*. *mercenaria*, two cases), Yesso scallops (*M*. *yessoensis*, two cases), and Mediterranean mussels (*M*. *galloprovincialis*, two cases). In contrast, OA-group toxins were not detected in non-bivalve samples (flatfish, conch, crab, sea cucumber, sea urchin, and abalone). The samples with the highest levels for each toxin were OA (18.1 µg/kg) in *M. coruscus*, DTX1 (40.5 µg/kg) in *M*. *coruscus*, and DTX3 (113.7 µg/kg) in *M*. *mercenaria* (Table 2).

**Table 1:** Occurrences of OA-group toxins in 217 seafood samples collected across South Korea

Parameter	OA	DTX1	DTX2	DTX3^a^
Total number of samples	217	217	217	217
Number of samples with a particular OA toxin (*n*)	5	9	0	20
Detection rate (%)	2.3	4.1	0	9.2
Positive mean (µg/kg)	11.3 ± 4.1	16.4 ± 11.0	0	40.9 ± 31.2
Minimum concentration (µg/kg)	7.36	8.41	ND^b^	12.85
Maximum concentration (µg/kg)	18.05	40.47	ND	113.69

OA, okadaic acid; DTX, dinophysistoxin

^a^Concentration of OA, DTX1, and DTX2 (with hydrolysis step) − concentration of OA, DTX1, and DTX2 (without hydrolysis step)

^b^Not detected (< limit of detection)

**Table 2:** Levels of OA-group toxins in bivalve and non-bivalve seafood samples collected across South Korea

Sample name	Species	*N*	Positive mean (µg/kg)					Range (µg/kg)			
			OA	DTX1	DTX2	DTX3		OA	DTX1	DTX2	DTX3
Bivalve											
Blood clam	*Anadara broughtonii*	13	10.4 (2)^a^	8.7 (2)	ND^b^	14.5 (2)		9.0–11.7	8.4–9.0	ND	13.3–15.8
Cockle	*Tegillarca granosa*	13	ND	ND	ND	ND		ND	ND	ND	ND
Clam	*Ruditapes philippinarum*	21	ND	ND	ND	32.9 (5)		ND	ND	ND	16.2–52.6
Fan shell	*Atrina pectinata*	18	ND	ND	ND	54.1 (2)		ND	ND	ND	41.5–66.6
Hard clam	*Mercenaria mercenaria*	26	ND	ND	ND	105.8 (2)		ND	ND	ND	97.9–113.7
Japanese cockle	*Fulvia mutica*	15	ND	ND	ND	ND		ND	ND	ND	ND
Mactra	*Mactra quadrangularis*	9	ND	ND	ND	ND		ND	ND	ND	ND
	*Mactra chinensis*	2	ND	ND	ND	ND		ND	ND	ND	ND
Mussel	*Mytilus coruscus*	5	11.9 (3)	21.2 (5)	ND	46.0 (5)		7.5–18.1	8.5–40.5	ND	12.9–73.8
	*Mytilus galloprovincialis*	16	ND	11.8 (2)	ND	1.6 (1)		ND	9.8–13.9	ND	1.6
Oyster	*Crassostrea gigas*	9	ND	ND	ND	ND		ND	ND	ND	ND
Scallop	*Mizuhopecten yessoensis*	14	ND	ND	ND	49.3 (2)		ND	ND	ND	13.5–85.0
	*Argopecten irradians*	9	ND	ND	ND	7.3 (1)		ND	ND	ND	7.3
Soft-shell clam	*Mya arenaria*	1	ND	ND	ND	ND		ND	ND	ND	ND
Sunset clam	*Megangulus venulosus*	1	ND	ND	ND	ND		ND	ND	ND	ND
Surf clam	*Spisula sachalinensis*	10	ND	ND	ND	ND		ND	ND	ND	ND
Non-bivalve abalone	*Haliotis discus hannai*	9	ND	ND	ND	ND		ND	ND	ND	ND
Conch	*Turbo cornutus*	1	ND	ND	ND	ND		ND	ND	ND	ND
	*Rapana venosa*	5	ND	ND	ND	ND		ND	ND	ND	ND
Crab	*Portunus trituberculatus*	4	ND	ND	ND	ND		ND	ND	ND	ND
Flatfish	*Paralichthys olivaceus*	6	ND	ND	ND	ND		ND	ND	ND	ND
Sea cucumber	*Apostichopus japonicus*	4	ND	ND	ND	ND		ND	ND	ND	ND
Sea urchin	*Heliocidaris crassispina*	6	ND	ND	ND	ND		ND	ND	ND	ND

OA, okadaic acid; DTX, dinophysistoxin

^a^Number of positive samples is in parentheses

^b^Not detected (< limit of detection)

**Fig. 1:**
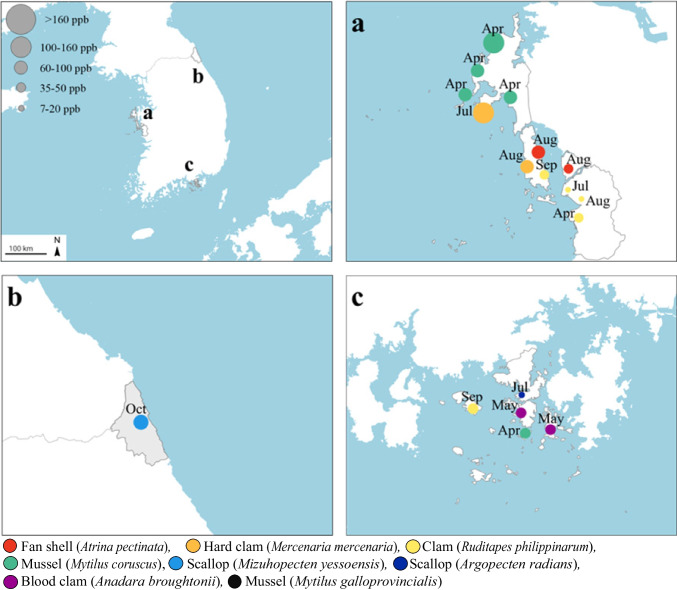
Detection of OA-group toxin distribution according to sampling sites and marine species. **a** Taean, Hongseong (36.56°N, 126.25°E, West Sea). **b** Goseong (38.41°N, 128.63°E, East Sea). **c** Tongyeong (34.82°N, 128.34°E, South Sea). Each colored circle represents a marine species, and its size represents the OA equivalent toxin concentration. OA, okadaic acid. The three letters around each colored circle indicate sampling time (Apr, April; Jul, July; Aug, August; Sep, September; Oct, October)

The OA-group toxins showed different trends in detection frequencies and levels depending on the sampling site (Fig. 1). In samples from the East Sea coastal area (*n* = 13, Goseong region; 38.41°N, 128.63°E), the detection rate and level of OA-group toxins were 8% and 85.03 µg OA eq./kg, respectively. In samples from the West Sea coastal area (*n* = 75, Taean and Hongseong regions; 36.56°N, 126.25°E), a detection rate of 16% and detection range of 16.17–121.73 µg OA eq./kg were observed. The samples from the South Sea coastal area (*n* = 97, Tongyeong region; 34.82°N, 128.34°E) exhibited a detection rate of 5% and detection range of 11.35–13.90 µg OA eq./kg. Among the imported samples (*n* = 32), OA-group toxins were detected in two cases of mussels (*M*. *galloprovincialis*) from China (detection range, 11.35–13.90 µg OA eq./kg) and in one case in scallops (*M*. *yessoensis*) from Japan (13.48 µg OA eq./kg). However, there was no statistical significance for OA group toxins depending on the collection location (three coastal areas) (*p* > 0.05).

**Fig. 2:**
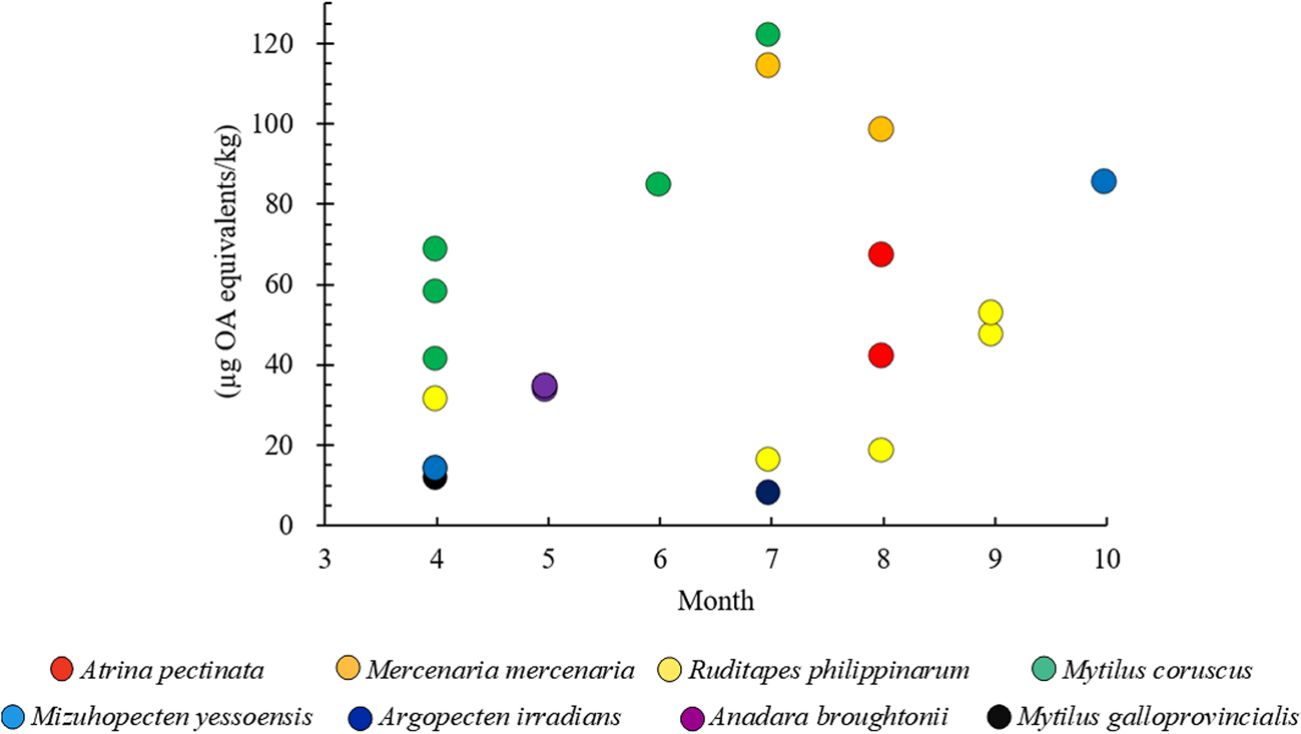
Monthly variation in OA-group toxins in seafood samples from South Korea during 2021. OA, okadaic acid

The detection rate of OA group toxins differed depending on the month. The monthly detection rate was 16% in April, 5% in May, 3% in June, 10% in July, 13% in August, 18% in September, and 17% in October. Excluding May and June, the monthly detection rates were all similar. However, although not statistically significant (*p* > 0.05), the levels detected in the summer months of June, July, and August tended to be higher than those observed in the other months. In July, the positive mean was 64.72 µg/kg, and the detection range was 7.27–121.73 µg/kg. In August, the positive mean was 55.99 µg/kg, and the detection range was 17.95–97.88 µg/kg (Fig. 2).

### Acute risk assessment of OA-group toxins

**Fig. 3:**
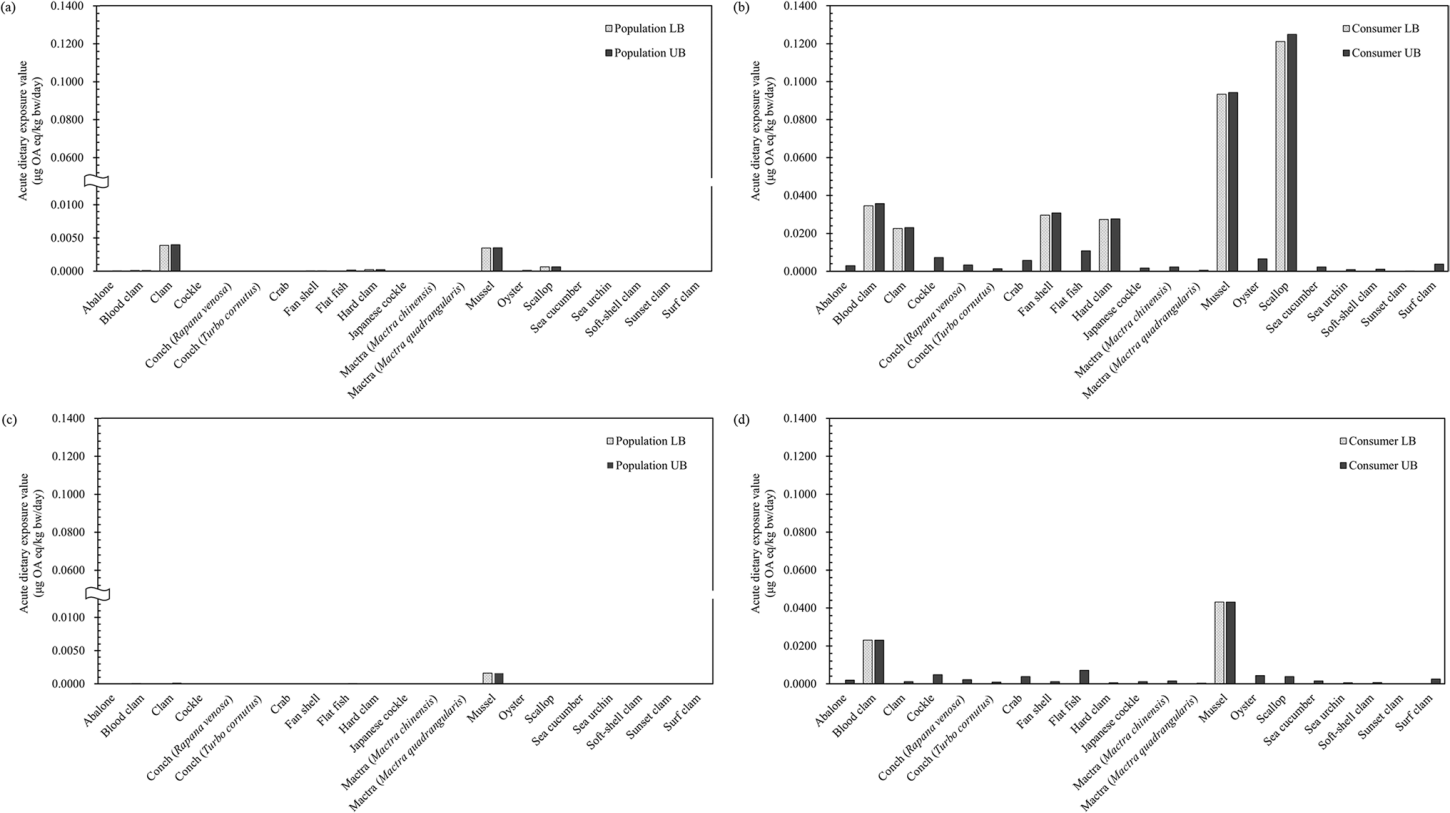
Acute dietary exposure estimates for the Korean population and consumer groups based on LB and UB occurrence data. **a** Acute dietary exposure estimate for the Korean population using occurrence data of the sum of OA, DTX1, DTX2, and DTX3. **b** Acute dietary exposure estimate for the consumer group using occurrence data of the sum of OA, DTX1, DTX2, and DTX3. **c** Acute dietary exposure estimate for the Korean population using occurrence data of the sum of OA and DTX1. **d** Acute dietary exposure estimate for the consumer group using occurrence data of the sum of OA and DTX1. OA, okadaic acid; DTX, dinophysistoxin; LB, lower bound; UB, upper bound

Figure 3 shows the acute dietary exposure values using LB and UB occurrence data for the sum of OA, DTX1, DTX2, and DTX3, and the sum of OA and DTX1 in the population groups (A and C) and consumer groups (B and D). The acute dietary exposure values in the population and consumer groups based on the sum of the occurrence of OA, DTX1, DTX2, and DTX3 are illustrated in Fig. 3A and B. The highest exposure values for the population were observed in clams, with LB and UB values of 0.0039 and 0.0040 μg OA eq./kg bw/day, respectively (Fig. 3A). These values corresponded to 1.18% (LB) and 1.21% (UB) of the ARfD (0.33 μg OA eq./kg bw/day) established by the FAO/IOC/WHO. In contrast, the highest exposure for the consumer group was observed in scallops, with LB and UB values of 0.1212 and 0.1250 μg OA eq./kg bw/day, respectively (Fig. 3B), which corresponded to 36.73% (LB) and 37.87% (UB) of the ARfD proposed by the FAO/IOC/WHO.

Figure 3C and D illustrate the acute dietary exposure values in the population and consumer groups based on the sum of the OA and DTX1 occurrence data. Overall, the exposure values based on the sum of the OA and DTX1 occurrence data were lower than those based on the sum of the OA, DTX1, DTX2, and DTX3 occurrence data. Mussels contributed to the highest exposure values among the population, with LB and UB values of 0.0016 μg OA eq./kg bw/day (Fig. 3C), which corresponded to 0.48% (LB and UB) of the ARfD (0.33 μg OA eq./kg bw/day). The highest exposure levels in the consumer groups were also observed among mussel consumers at 0.0431 μg OA eq./kg bw/day (LB and UB; Fig. 3D), which corresponded to 13.1% (LB and UB) of the ARfD.

**Fig. 4:**
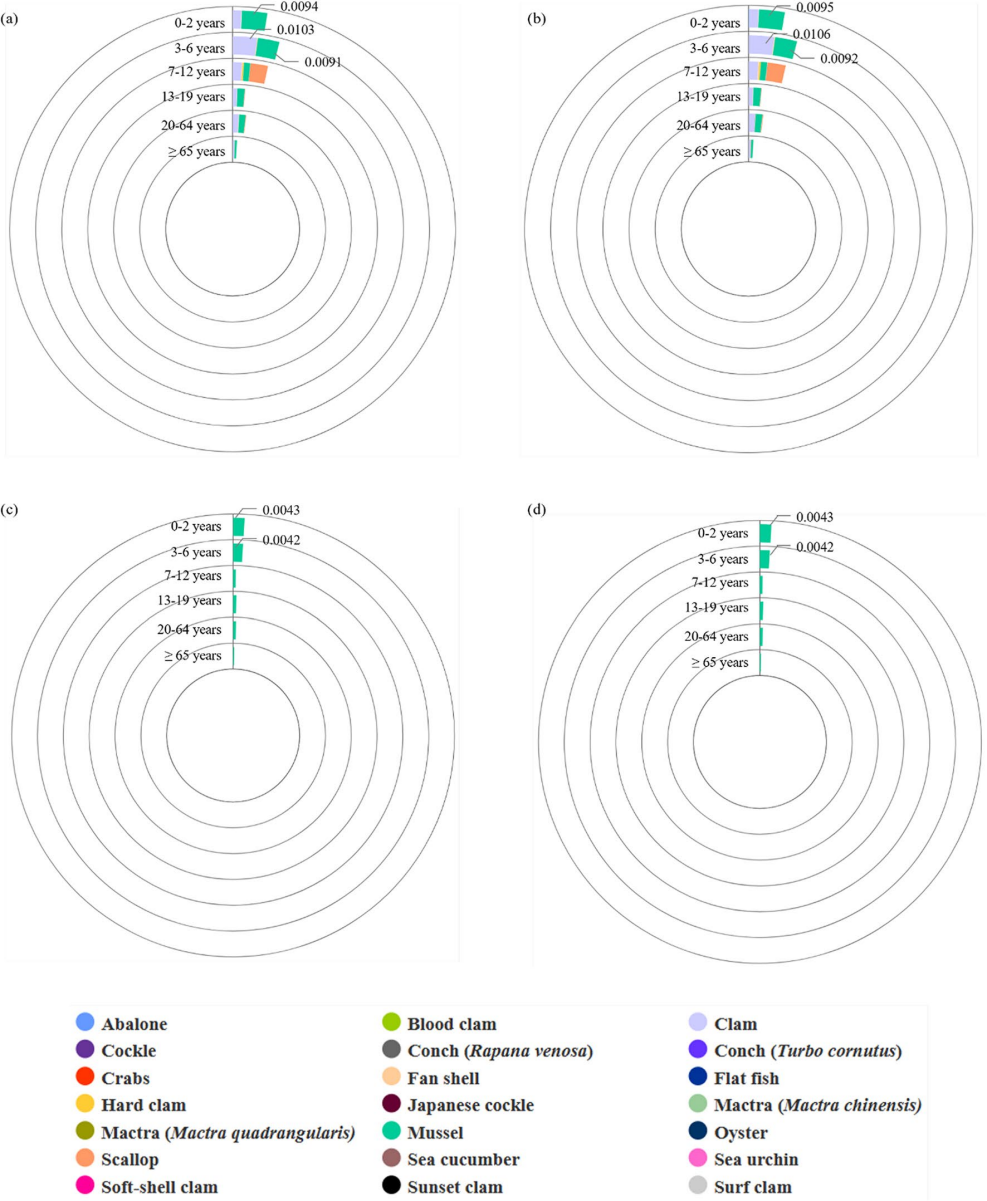
Acute dietary exposure values (μg OA eq/kg bw/day) in population groups by age. **a** Occurrence (LB) for the sum of OA, DTX1, DTX2, and DTX3. **b** Occurrence (UB) for the sum of OA, DTX1, DTX2, and DTX3. **c** Occurrence (LB) for the sum of OA and DTX1. **d** Occurrence (UB) for the sum of OA and DTX1. OA, okadaic acid; DTX, dinophysistoxin; LB, lower bound; UB, upper bound; eq, equivalent; bw, body weight

Figure 4 shows the acute dietary exposure values by age in the Korean population using LB and UB occurrence data for the sum of OA, DTX1, DTX2, and DTX3 (A and B), and the sum of OA and DTX1 (C and D). According to the results by age using the sum of OA, DTX1, DTX2, and DTX3 (Fig. 4A and B), the exposure values of 3–6-year-old children who consumed clams in the Korean population were the highest among all bivalve and non-bivalve samples, with LB and UB values of 0.0103 and 0.0106 μg OA eq./kg bw/day, respectively. According to the results by age using the sum of OA and DTX1 (Fig. 4C and D), the exposure value of 0–2-year-old children who consumed mussels was the highest among all bivalve and non-bivalve samples at 0.0043 μg OA eq./kg bw/day (LB and UB). These exposure values did not exceed the ARfDs.

**Fig. 5:**
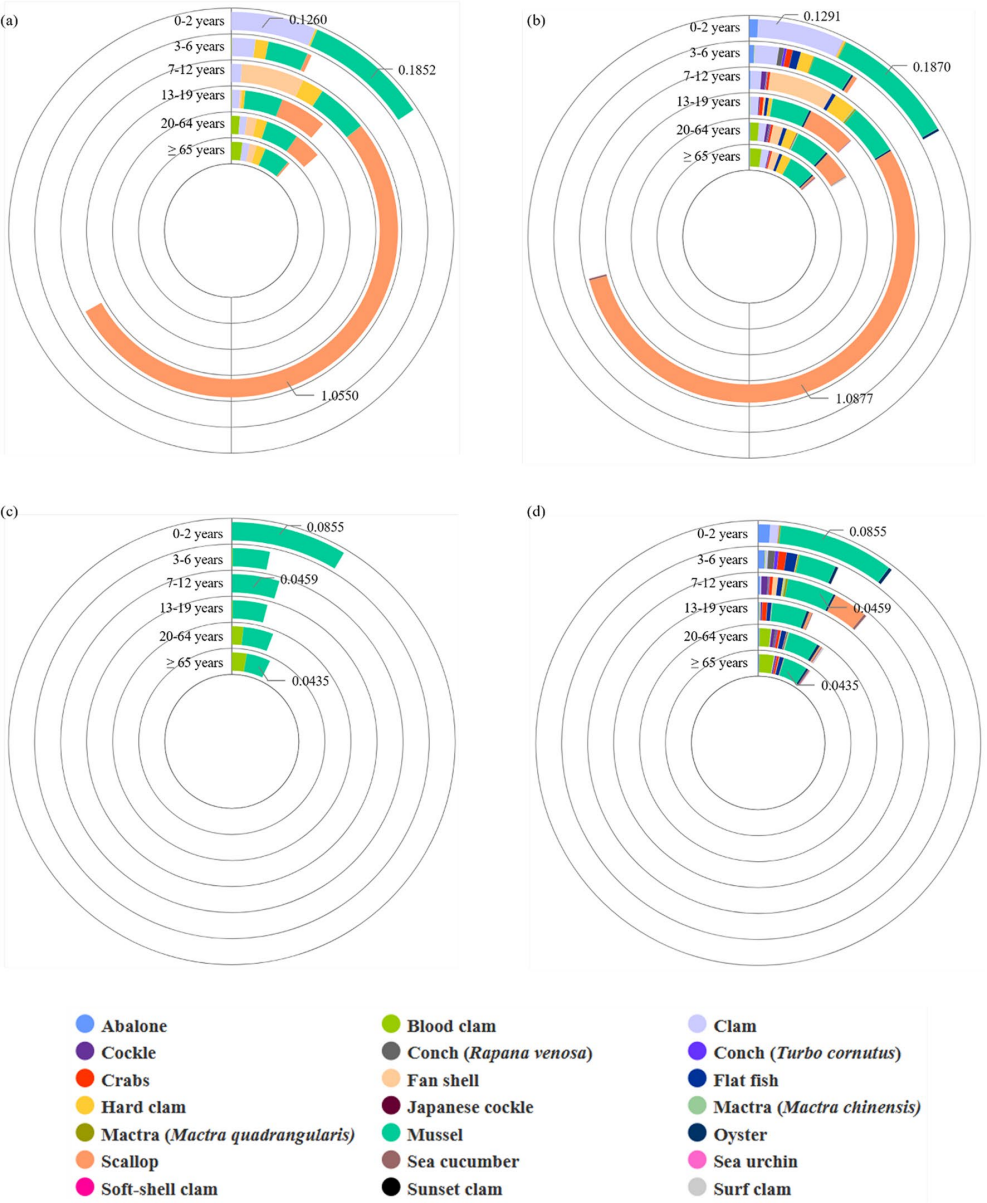
Acute dietary exposure values (μg OA eq/kg bw/day) in consumer groups by age. **a** Occurrence (LB) for the sum of OA, DTX1, DTX2, and DTX3. **b** Occurrence (UB) for the sum of OA, DTX1, DTX2, and DTX3. **c** Occurrence (LB) for the sum of OA and DTX1. **d** Occurrence (UB) for the sum of OA and DTX1. OA, okadaic acid; DTX, dinophysistoxin; LB, lower bound; UB, upper bound; eq, equivalent; bw, body weight

Figure 5 shows the acute dietary exposure values in the consumer groups by age using LB and UB occurrence data for the sum of OA, DTX1, DTX2, and DTX3 (A and C), and the sum of OA and DTX1 (B and D). Among the consumer groups using occurrence data for the sum of OA, DTX1, DTX2, and DTX3 (Fig. 5A and C), the highest exposure occurred in individuals 7–12 years of age who consumed scallops, with LB and UB values of 1.0550 and 1.0877 μg OA eq./kg bw/day, respectively. These values exceeded the ARfDs established by the FAO/IOC/WHO. According to the results by age using the sum of OA and DTX1 (Fig. 5C and D), the acute exposure value in the consumer groups was the highest among individuals 0–2 years of age who consumed mussels, with exposure values of 0.0855 μg OA eq./kg bw/day (LB and UB). Unlike the exposure values using occurrence data for the sum of OA, DTX1, DTX2, and DTX3, the exposure values based on the occurrence data for the sum of OA and DTX1 did not exceed the ARfDs.

Overall, we observed clear differences in the estimates of acute OA-group toxin exposure by age depending on whether the calculations were based on the sum of the OA and DTX1 occurrence data or the sum of the OA, DTX1, DTX2, and DTX3 occurrence data. The acute exposure values estimated using the latter data resulted in higher values.

## Discussion

In the present study, the occurrence of OA-group toxins, including OA, DTX1, DTX2, and DTX3, in seafood samples obtained from South Korea was analyzed using LC-MS/MS, and risk assessment was conducted based on the seafood consumption trends in the Korean population. This study explored the occurrence of OA, DTX1, and DTX2 and their acylated form, DTX3, among bivalves and non-bivalves distributed across Korea. Additionally, based on the occurrence data used to conduct the risk assessment analyses, we identified a potential health risk due to acute exposure to OA-group toxins for scallop consumers aged 7 to 12 years. These results demonstrated that the inclusion of DTX3 in the new regulatory limits is appropriate to protect Korean seafood consumers from exposure to OA-group toxins.

Most recent OA-group toxin studies have focused on major bivalves such as mussels and oysters (Bazzoni et al. 2018; Huang et al. 2014; Kilcoyne and Fux 2010; Rossignoli et al. 2021; Schirone et al. 2018). However, OA-group toxins can be detected in other bivalves such as clams, cockles, and scallops (Blanco 2018). Additionally, OA-group toxins have been detected in fish that consume bivalves such as halibut and anchoveta (Corriere et al. 2021; Costa et al. 2017). In the present study, however, OA-group toxins were only detected in bivalve samples. Among these, *M*. *coruscus* was highly contaminated with OA, DTX1, and DTX3 levels. In contrast, no OA-group toxins were detected in non-bivalve samples (flatfish, conch, crab, sea cucumber, sea urchin, and abalone). These results confirm that toxin accumulation occurs more frequently in filter-feeding bivalves (Blanco 2018). Relatively higher concentrations of OA group toxins were detected from the samples collected in summer (June, July, and August), although the differences among months were not statistically significant (*p* > 0.05). This appears to be at least partially related to the high sea temperature of the Korean Peninsula in June, July, and August (Korea Meteorological Administration 2021). However, because the occurrence of OA group toxins is complexly influenced by various marine geo-ecological factors, an additional study is needed to determine the exact reason.

This study demonstrated that DTX3 contributed significantly to the level and incidence of OA-group toxins in seafood distributed across Korea. This is consistent with previous studies in which DTX3 was reported to contribute considerably to the total content of OA-group toxins (EFSA 2008; Hossen et al. 2011). Among OA, DTX1, DTX2, and DTX3, DTX3 was the most prevalent toxin, with a positive mean level of 113.7 µg/kg, followed by DTX1 and OA. DTX3 represents a group of fatty acid ester derivatives produced by the acylation of OA, DTX1, and DTX2 in the digestive gland of shellfish (Lee et al. 2016; Yasumoto et al. 1985). In particular, DTX3 was also detected in bivalves collected from the coastal area of the East Sea, where OA-group toxins had not previously been detected (Lee et al. 2011) because the seawater temperature was relatively low and located relatively north (38.41°N, 128.63°E). DTX3 is known to be less toxic than its parent toxins because of conformational changes that disrupt binding to PPs (McNabb 2008). However, DTX3 has the same toxic potential as the parent toxins because it is metabolically deacylated into the parent toxin in the human stomach after consumption of contaminated seafood (García et al. 2005). Kameneva et al. (2015) found that OA, DTX1, and DTX2 were mostly concentrated in the digestive gland of *Crenomytilus grayanus* (60–70%), whereas DTX3 was more abundant in the edible soft tissues (> 80%). These results indicate that DTX3 should be monitored and included in the current regulatory limits to minimize dietary exposure to OA-group toxins in seafood distributed across Korea.

DTX2 is an isomer of OA with a methyl group on carbon 35 but not on carbon 31 (Hu et al. 1992). *D*. *acuta* and *D*. *caudata*, both of which are representative DTX2-producing microalgae, were detected recently in the Korean South Sea (Park et al. 2019). However, DTX2 was not detected in the 217 bivalve and non-bivalve samples collected in the current study. In contrast, DTX2 has been detected in shellfish from Ireland, Spain, Portugal, and Norway (Carmody et al. 1996; Gago-Martinez et al. 1996; Hu et al. 1992; Vale et al. 1998). High amounts of DTX2 have also been detected in shellfish from the Galician region of Spain (Blanco et al. 1995) and in Portuguese mussels (Vale and Sampayo 1996; Vale et al. 2008). Moreover, DTX1, also known as 35-methyl OA, is the dominant toxin in Japan and neighboring Korea (Lee et al. 1989; Kim et al. 2008; Yasumoto et al. 1985). Consistent with these findings, DTX1 was detected in 4% of seafood samples in our study. OA occurs worldwide but is reported to be the dominant OA-group toxin in Europe (FAO/WHO 2016). Although OA did not represent a large proportion of the OA-group toxins examined in this study, 2.3% of the samples still exhibited a positive mean level of 11.3 µg/kg. Presumably, this difference in the occurrence of OA-group toxins is at least partially related to marine geo-ecological factors such as salinity, sunlight, temperature, oxygen, pH, wind or water currents, soil type, and nutrient availability (Borges et al. 2022; Griffith and Gobler 2020; Hallegraeff 2010). However, other factors such as insufficient sample size or the brevity of our study period cannot be excluded. Therefore, additional research is needed to clarify the degree to which the sample size and study duration influenced our results. Since toxic *Dinophysis* species including *D. acuminata*, *D. acuta*, and *D. caudata* were found in the Korean coast (Kang and Lee 2018; Park et al. 2019), systematic and continuous monitoring of OA group toxins is necessary.

Our risk assessment analysis using the occurrence level data (sum of OA, DTX1, DTX2, and DTX3) revealed that the intoxication risk in the Korean population and consumer groups was generally low. Among the OA-group toxins, the relative exposure contributions of individual OA, DTX1, and DTX3 to total dietary exposure were 8.3%, 16.9%, and 74.8%, respectively (data not shown). By age, the acute dietary exposure values of children were not low. Particularly, the dietary exposure to OA-group toxins among 7–12-year-old consumers of scallops was 1.0877 µg OA eq./kg bw/day. This may be attributed to the high food consumption rates in this age group coupled with a substantially lower body mass. This observed value exceeded the ARfD, indicating that scallop consumption among 7–12-year-olds may cause diarrhea symptoms. However, only 12 survey respondents in the 7–12 age group consumed scallops, and therefore, these results should be interpreted cautiously.

Upon comparing the occurrence data for the sum of OA, DTX1, DTX2, and DTX3 with those for the sum of OA and DTX1, the exposure values in the former dataset were 1.0550 (LB) and 1.0877 (UB) µg OA eq./kg bw/day in the 7–12 age group that consumed scallops, which exceeded the ARfD by 3.2- to 3.6-fold. However, in the latter dataset, the exposure values were 0.0000 (LB) and 0.0003 (UB) µg OA eq./kg bw/day, which did not exceed the ARfD. This occurred because, unlike DTX3, OA and DTX1 were not detected. Therefore, to protect consumers from exposure to OA-group toxins, the regulation that uses the sum of OA and DTX1 should be changed to one that accounts for all OA-group toxins, including OA, DTX1, DTX2, and DTX3.

The EFSA has also conducted DSP risk assessments in the context of acute OA-group toxin exposure. In that risk assessment, the acute dietary exposure value was derived from 96 µg of toxin (equivalent to 1.6 µg/kg bw in a 60 kg adult) (EFSA 2008), which was approximately 20 × higher than the maximum value of 0.0801 OA eq. µg/kg bw/day (the scallop value for the 20–64-year-old consumer group based on UB data) in the > 20-year-old adult group in our study. In another study conducted by New Zealand Food Safety, the exposure value was 1.07 µg OA eq./kg bw/day at the maximum level in adults when samples exceeding the regulatory limit for DSP were excluded. However, when these samples were included, the exposure value was 9.43 µg OA eq./kg bw/day (New Zealand Ministry for Primary Industries 2020). Moreover, these values were higher than the results for the adult group in our study.

It is important to note that the risk assessment conducted herein has some limitations, including the size of the sample occurrence dataset, the effect of cooking and processing, and the accuracy of the consumption data. A more realistic risk assessment could be achieved using a probabilistic approach (considering the distribution of all data, both contamination and consumption). However, this approach requires more data on the occurrence of OA-group toxins in shellfish (Anses 2019). Additionally, acute risk assessment is performed based on an average shellfish portion size in most countries and agencies, including the EFSA. Given that such portion sizes have not been established for the Korean population, the risk assessment results of other countries cannot be easily compared with those determined herein. Therefore, calculating a shellfish portion size is crucial for future risk assessments to ensure the safety of Korean consumers.

## Conclusion

The occurrence of OA-group toxins was examined in 217 samples of South Korean seafood, encompassing 16 bivalve and seven non-bivalve species collected from three representative coastal areas. Among the examined OA-group toxins, OA, DTX1, and DTX3 were detected in the seafood samples, and DTX3 was the most prevalent toxin in South Korean bivalve samples. In particular, DTX3 was also detected in bivalves collected from the coastal area of the East Sea, where OA-group toxins had not previously been detected. The bivalve samples in which at least one OA-group toxin was detected were blood clam, pan shell, hard clam, mussel, and scallop; no toxins were detected in non-bivalves.

Our risk assessment results suggested that scallops and mussels are the major contributors to OA-group toxin exposure in the Korean population and consumer groups. Regarding scallops and mussels, monitoring at the production stage should be strengthened to prevent seafood with OA-group toxin levels exceeding the regulatory limit from entering the distribution stage. Regarding age, relatively high dietary exposure values were found in children. Therefore, seafood consumption by children should be monitored more closely. Until 2023, South Korea had set a regulatory limit for free OA and DTX1, which was ≤ 160 μg OA eq./kg (MFDS 2016). To protect consumers from exposure to OA-group toxins, regulatory strategies must account for all OA-group toxins, including OA, DTX1, DTX2, and DTX3, instead of only considering the sum of OA and DTX1.

### Supplementary Information

Below is the link to the electronic supplementary material.

Supplementary file1 (DOCX 47 KB)

## Data Availability

The data sets used and analyzed during the current study are available from the corresponding author upon reasonable request and after obtaining permission from the funding agency.
