# Supplementary material for: Occurrence and risk assessment of okadaic acid, dinophysistoxin-1, dinophysistoxin-2, and dinophysistoxin-3 in seafood from South Korea
Source: Environ Sci Pollut Res Int. 2023 Dec 26;31(4):6243–57. doi: 10.1007/s11356-023-31568-4 (PMC10799158; doi:10.1007/s11356-023-31568-4)
Supplement: Supplementary file 1 — Supplementary file1 (DOCX 47 KB) [file 11356_2023_31568_MOESM1_ESM.docx]

Supplementary Table S1. Seafood samples collected in 2021 in Korea.

| **Sample**  **name** | **Species** |  | **Sampling** **site**  **(origin)** |  | **Sampling month** | | | | | | |
| --- | --- | --- | --- | --- | --- | --- | --- | --- | --- | --- | --- |
|  |  |  |  |  | **Apr.** | **May** | **Jun.** | **Jul.** | **Aug.** | **Sep.** | **Oct.** |
| Abalone | *Haliotis discus*  *hannai* |  | Tongyeong (South Sea) |  | 3 | -^a^ | 2 | 2 | 2 | - | - |
| Blood clam | *Anadara*  *broughtonii* |  | Tongyeong (South Sea) |  | 3 | 8 | 2 | - | - | - | - |
| Cockle | *Tegillarca*  *granosa* |  | Beolgyo (South Sea) |  | 2 | 8 | 2 | 1 | - | - | - |
| Conch | *Turbo*  *cornutus* |  | Boryeong (South Sea) |  | 1 | - | - | - | - | - | - |
|  | *Rapana*  *venosa* |  | Jeju (West Sea) |  | - | - | 2 | 2 | 1 | - | - |
| Clam | *Ruditapes*  *philippinarum* |  | Hongseong (West Sea) |  | 3 | - | 2 | 2 | 2 | 1 | 1 |
|  |  |  | Tongyeong (South Sea) |  | 4 | - | - | 2 | 2 | 1 | 1 |
| Crab | *Portunus*  *trituberculatus* |  | Taean (West Sea) |  | - | 4 | - | - | - | - | - |
| Fan shell | *Atrina*  *pectinata* |  | Hongseong (West Sea) |  | 2 | - | 2 | 2 | 2 | - | - |
|  |  |  | Tongyeong (South Sea) |  | 2 | - | 2 | 2 | 2 | 1 | 1 |
| Flat fish | *Paralichthys*  *olivaceus* |  | Seocheon (West Sea) |  | - | - | 2 | 2 | 2 | - | - |
| Hard clam | *Mercenaria*  *mercenaria* |  | Taean (West Sea) |  | 1 | 6 | 2 | 2 | 2 | 1 | 1 |
|  |  |  | Pohang (South Sea) |  | 2 | - | - | - | - | - | - |
|  |  |  | Online (China) |  | 2 | 2 | 1 | 2 | 1 | 1 | - |
| Japanese  cockle | *Fulvia*  *mutica* |  | Hongseong (West Sea) |  | 2 | 3 | - | - | - | - | - |
|  |  |  | Yeosu (South Sea) |  | 2 | 8 | - | - | - | - | - |
| Mactra | *Mactra*  *chinensis* |  | Boryeong (West Sea) |  | - | 2 | - | - | - | - | - |
|  | *Mactra*  *quadrangularis* |  | Gochang (West Sea) |  | 2 | - | 2 | 2 | 2 | 1 | - |
| Mussel | *Mytilus*  *coruscus* |  | Taean (West Sea) |  | 3 | - | 1 | 1 | - | - | - |
|  | *Mytilus*  *galloprovincialis* |  | Tongyeong (South Sea) |  | 2 | - | 2 | 2 | 2 | 1 | 1 |
|  |  |  | Online (imported from China) |  | 2 | - | - | 2 | 2 | - | - |
| Oyster | *Crassostrea*  *gigas* |  | Tongyeong (South Sea) |  | 3 | - | 2 | 2 | 2 | - | - |
| Scallops | *Mizuhopecten*  *yessoensis* |  | Goseong (East Sea) |  | 2 | - | 2 | - | - | - | 1 |
|  |  |  | Online (imported from Japan) |  | 2 | - | 2 | 2 | 2 | 1 | - |
|  | *Argopecten*  *irradians* |  | Tongyeong (South Sea) |  | 3 | - | 2 | 2 | 1 | 1 | - |
| Sea cucumber | *Apostichopus*  *japonicus* |  | Boryeong (West Sea) |  | - | - | 2 | 2 | - | - | - |
| Sea urchin | *Heliocidaris*  *crassispina* |  | Yeosu (East Sea) |  | - | - | 2 | 2 | 2 | - | - |
| Soft-shell clam | *Mya*  *arenaria* |  | Tongyeong (West Sea) |  | 1 | - | - | - | - | - | - |
| Sunset clam | *Megangulus*  *venulosus* |  | Pohang (South Sea) |  | 1 | - | - | - | - | - | - |
| Surf clam | *Spisula*  *sachalinensis* |  | Goseong (East Sea) |  | - | - | - | 1 | - | 1 | - |
|  |  |  | Online (imported from Russia) |  | 2 | - | 2 | 2 | 1 | 1 | - |
|  | Total |  |  |  | 52 | 41 | 38 | 39 | 30 | 11 | 6 |

No sample was collected.

Supplementary Table S2. Linearity, the limit of detection (LOD), the limit of quantification (LOQ), recovery accuracy, and precision of OA-group toxins in three matrices.

| **Matrix** | **Toxin** | **Range (µg/kg)** | **RSQ** | **LOD (µg/kg)** | **LOQ (µg/kg)** |  | **Spiking concentration (µg/kg)** | | **Recovery (%) ± RSD (%)** | | |  | **Recovery (%) ± RSD (%)** | | |
| --- | --- | --- | --- | --- | --- | --- | --- | --- | --- | --- | --- | --- | --- | --- | --- |
|  |  |  |  |  |  |  |  |  | **Intra-day** | | |  | **Inter-day** | | |
| Flatfish | OA | LOD-40 | 0.999 | 2.0 | 6.0 |  | | 100 | 105.7 | **±** | 5.8 |  | 98.7 | **±** | 7.7 |
|  | DTX1 |  | 0.998 | 2.6 | 7.8 |  | | 100 | 82.9 | **±** | 4.1 |  | 98.6 | **±** | 14.6 |
|  | DTX2 |  | 0.999 | 0.7 | 2.2 |  | | 100 | 89.8 | **±** | 5.6 |  | 94 | **±** | 4.8 |
| Clam | OA | LOD-40 | 0.999 | 2.1 | 6.2 |  | | 100 | 85.7 | **±** | 7.1 |  | 92.3 | **±** | 8.6 |
|  | DTX1 |  | 0.997 | 0.5 | 1.6 |  | | 100 | 99.2 | **±** | 7.7 |  | 87.8 | **±** | 11.3 |
|  | DTX2 |  | 0.997 | 2.6 | 7.8 |  | | 100 | 91.8 | **±** | 17.0 |  | 94.9 | **±** | 2.9 |
| Mussel | OA | LOD-40 | 0.999 | 0.5 | 1.6 |  | | 100 | 93.9 | **±** | 3.9 |  | 91.0 | **±** | 4.6 |
|  | DTX1 |  | 0.999 | 0.5 | 1.4 |  | | 100 | 92.8 | **±** | 3.9 |  | 90.4 | **±** | 3.8 |
|  | DTX2 |  | 0.999 | 0.4 | 1.2 |  | | 100 | 109.3 | **±** | 2.0 |  | 98.7 | **±** | 15.3 |
